# Parsing Neurodynamic Information Streams to Estimate the Frequency, Magnitude and Duration of Team Uncertainty

**DOI:** 10.3389/fnsys.2021.606823

**Published:** 2021-02-01

**Authors:** Ronald H. Stevens, Trysha L. Galloway

**Affiliations:** ^1^University of California Los Angeles (UCLA) School of Medicine, Brain Research Institute, Culver City, CA, United States; ^2^The Learning Chameleon, Inc., Culver City, CA, United States

**Keywords:** teamwork, EEG, uncertainty, information, team neurodynamics, social coordination dynamics

## Abstract

Neurodynamic organizations are information-based abstractions, expressed in bits, of the structure of long duration EEG amplitude levels. Neurodynamic information (*NI*, the variable of neurodynamic organization) is thought to continually accumulate as EEG amplitudes cycle through periods of persistent activation and deactivation in response to the activities and uncertainties of teamwork. Here we show that (1) Neurodynamic information levels were a better predictor of uncertainty and novice and expert behaviors than were the EEG power levels from which *NI* was derived. (2) Spatial and temporal parsing of team N*I* from experienced submarine navigation and healthcare teams showed that it was composed of discrete peaks with durations up to 20–60 s, and identified the involvement of activated delta waves when precise motor control was needed. (3) The relationship between *NI* and EEG power was complex varying by brain regions, EEG frequencies, and global vs. local brain interactions. The presence of an organizational system of information that parallels the amplitude of EEG rhythms is important as it provides a greatly reduced data dimension while retaining the essential system features, i.e., linkages to higher scale behaviors that span temporal and spatial scales of teamwork. In this way the combinatorial explosion of EEG rhythmic variables at micro levels become compressed into an intermediate system of information and organization which links to macro-scale team and team member behaviors. These studies provide an avenue for understanding how complex organizations arise from the dynamics of underlying micro-scale variables. The study also has practical implications for how micro-scale variables might be better represented, both conceptually and in terms of parsimony, for training machines to recognize human behaviors that span scales of teams.

## Introduction

It has been proposed that biological systems, like teams, are hierarchies of information that are functionally organized across spatial and time scales (Flack, 2017a). Uncertainty is the messenger on this hierarchy guiding information back and forth between the environment and the team (Flack et al., 2012), with ripples and islands in these information streams representing periods of changing organization [see Stevens et al. (2013) for team examples]. This changing information helps the brain identify regularities in the environment and use them to shape adaptations along the macroscopic and microscopic continuum of experience and learning (Daniels et al., 2017).

In this process, uncertainty plays a unifying role in how the task and environment are perceived by a team (Stroulia and Goel, 1994; Hong, 2010), and how adaptive inference guides attention, motor control, decision making and ultimately learning (Friston et al., 2006; Friston, 2010; Sengupta et al., 2013; Dragomir et al., 2020).

As the brain learns to track patterns during training, discrete segments of cognitive stability develop that are energetically the “best fits” for predictively navigating through the tasks while avoiding surprise (Knill and Pouget, 2004). With practice, the responses and cognition required to execute them become more automatic, and require less energy to perform (Dunst et al., 2014; Sun et al., 2020). As a result we think and act in terms of schemata (Rumelhart, 1980), scripts (Schank and Abelson, 1977), chunks (Schneider and Logan, 2015), policies (Pezzulo et al., 2018) and episodes (Farooqui and Manly, 2018, 2019), which help streamline the moment to moment activities by structuring them into routines around frequently experienced events (Cooper and Shallice, 2000); in other words, exploiting what has already been experienced and learned (Daw et al., 2006). To the extent that the planning and execution of these routines meets the task requirements, the evolving situation will be predictable and teams will avoid surprise.

Occasionally, escalating unfamiliar environments and variable task outcomes generate unexpected sensory signals increasing uncertainty about what action to take next. This requires a switch from exploiting what has been learned to exploring and learning alternative approaches. The open-ended nature of exploration makes it difficult to predict how long the uncertainty will last (O'Rielly, 2013; Soltani and Izquierdo, 2019; Domenech et al., 2020), as the micro-states of uncertainty begin to scale into macro-states of pauses and hesitation (Kaufman et al., 2015). An open question, with implications for training to perform in complex environments, is how uncertainty at the neural level scales into observable hesitation.

Bottom up approaches to this question are complicated in that most low-level neural processes are not in themselves directly causal to team performance but involve everyday cognitive activities that support seeing, listening, decision making, etc. It is when these activities are transiently amplified or modified by the context, that they assume greater importance for teams. Studies performed at the scale of a half to one second have used EEG, fMRI, fNIRS or MRI and have measured the interpersonal dynamics of seen or heard motor actions (Caetano et al., 2007); speaker-listener coupling (Stephens et al., 2010; Perez et al., 2019); duets playing guitar (Sänger et al., 2012); and synchronic vs. diachronic movements (Tognoli and Kelso, 2015). These and other events are increasingly being probed by new sensor technologies and methods (Likens and Wiltshir, 2020).

The meaning of EEG power is also important. Alpha band oscillations (8–12 Hz) emphasize different functional properties depending on whether their states are synchronized (a.k.a. activated or high power) or desynchronized (a.k.a. deactivated or low power). Low power (i.e., often called suppressed) states are seen during attentive reading (Lachaux et al., 2008) and tend to favor new memory encodings. Higher alpha power states may transiently suppress gamma rhythms and help protect the contents of working memory from being disturbed thereby enhancing retention (Klimesch et al., 2006; Ossandon et al., 2011; Klimesch, 2012; Bonnefond and Jensen, 2015; Wianda and Ross, 2019). Similar considerations might apply to gamma waves (Sedley and Cunningham, 2013) and delta waves (1–4 Hz), which show an increase in power during the onset of fatigue, which decrease following challenge interruptions (Bodala et al., 2018).

There is a need however for higher level representations where modifications to and amplifications of the micro-scale dynamics are allowed to change freely, while still providing “best fit” (i.e., more stable) functional approximations for higher level activities (Nikolaus et al., 2008). In other words, abstractions that have a basis in mechanism but where many of the micro details don't need specifying (Flack, 2017b).

We have proposed that the informational properties of EEG rhythms might be candidates for such a representation as their basis in organization, not power or phase, may be more likely to align with processes responsible for observable macro-scale team organizations and behaviors. Such neurodynamic organizations contribute properties to the system not always possessed by the amplitude or phase of brainwaves. For instance, neurodynamic information (the variable of neurodynamic organization) has been shown to link with the organization of team activities (Stevens and Galloway, 2017) or speech (Gorman et al., 2015), or of team uncertainty (Stevens et al., 2016; Stevens and Galloway, 2019).

In this paper we parse the neurodynamics of teams into spatial and temporal subsets and merge them with lower level properties of EEG amplitude. The goal is to develop a framework for describing periods of neurodynamic uncertainty that could guide real-time feedback during training as well as support machines in their understanding of human uncertainty (Stevens and Galloway, 2019).

We consider three questions within this context.

1. Performing attention-demanding cognitive tasks requires not only regional activations but also deactivations that reflect decreased neural activity in regions supporting processes unrelated or irrelevant to the task at hand (i.e., Gusnard and Raichle, 2001; McKiernan et al., 2003; Fox et al., 2005; Lachaut et al., 2012). The first question asks whether there are periods of uncertainty, as identified by elevated neurodynamic organization, preferentially associated with periods of activated or deactivated EEG power? Are there channel or frequency dependencies for those periods where increased *NI* represents high EEG power vs. when they are represented by low EEG power?
2. Sensory likelihood functions, currently felt to be the lowest level where uncertainty is represented in cortical neurons are often unimodal (Beck et al., 2008; Walker et al., 2020) while multi-channel, multi-frequency-based team neurodynamic data streams appear multifractal (Likens et al., 2014). The second question asks whether the neurodynamic organizations associated with uncertainty are discrete at the sensor and frequency level or distributed across scalp regions and EEG frequencies?
3. Scalp-averaged *NI* levels vary based on their frequency, magnitudes and durations of neurodynamic organization. From a training and feedback perspective important questions are: How frequently does uncertainty occur? What is the level of uncertainty? How long is the uncertainty likely to last? The third question asks whether it is possible to develop quantitative neurodynamic models of the frequency, magnitude and duration of periods of uncertainty?

To expand the generality of the findings, examples include required US Navy submarine simulation training by experienced navy team members, required simulation training for experienced emergency air medical healthcare teams and advanced placement problem solving teams.

## Methods

### Teams and Tasks

**Figure 1:**
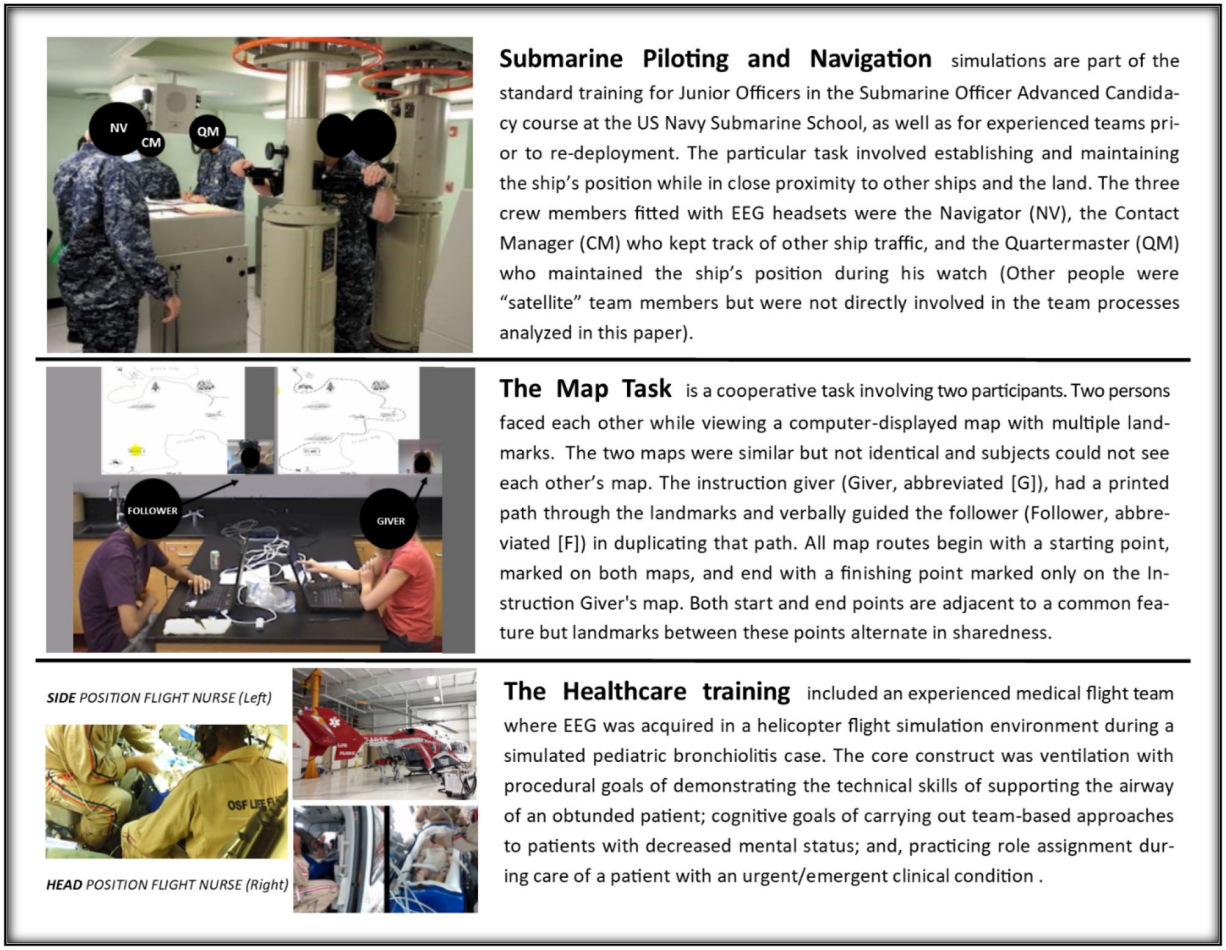
Summaries of the tasks and brief displays of their physical settings.

The data sets for these studies were sampled from teams performing complex and relevant tasks including (a) submarine navigation teams performing required simulations, (b) experienced and novice healthcare teams practicing patient ventilation, and c) advanced placement teams engaged in dyadic problem solving. The subjects, and task details for the Map Navigation Task (Stevens and Galloway, 2014, 2015), healthcare (Stevens et al., 2016, 2018; Stevens and Galloway, 2017) and Submarine Piloting Navigation (Stevens and Galloway, 2016, 2017; Stevens et al., 2017) have been previously described (Stevens and Galloway, 2017, 2019; Stevens et al., 2019). The physical settings and a brief summary of each task are shown in Figure 1.

### Ethics Statement

Informed consent protocols were approved by the Biomedical IRB, San Diego, CA (Protocol EEG01), the Order of Saint Francis Healthcare Institutional Review Board, and the Naval Submarine Medical Research Laboratory Institutional Review Board. All participating subjects consented (including images and speech for additional analysis) per approved applicable protocols. To maintain confidentially, each subject was assigned a unique number known only to the investigators of the study, and subject identities were not shared. This design complies with DHHS: protected human subject 45 CFR 46; FDA: informed consent 21 CFR 50.

### Modeling Neurodynamic Organizations and Information

The EEG sensors were adjusted for good contact (<10 Ω) and the EEG data streams (software timing with hardware timing) were synchronized with electronic time markers (e.g., trigger) as well as with (e.g., lab streaming layer, a.k.a. LSL) the events observed in audio/videos with <50 ms of accuracy (Kothe, 2014). Commonly found artifacts occur from speech, eye blinks, heartbeats, breathing rhythms and other electromyography sources. The pre-processing protocol for the EEG data streams were optimized for removing these artifacts. This included: (1) the rejection of bad channels, (2) using separate high and low pass frequency filters for detrending the data to properly calibrate the thresholds prior to detecting and adaptively removing sinusoidal noise (Mullen, 2012), enhanced source separation techniques (Delorme et al., 2007) for detecting artifacts and interpolating artifact “bursts” (Mullen et al., 2015) and (3) robust average referencing (Bigdely-Shamlo et al., 2015). Software tools included: customized MATLAB® scripts as well as the MATLAB®-based FieldTrip® toolbox (Oostenveld et al., 2011), the open source EEGLAB signal processing environment (Delorme and Makeig, 2004), Lab (ABM, Carlsbad) and/or, NeuroPype® Suite (Intheon Labs, San Diego) as previously reported (Stevens and Galloway, 2015, 2017, 2019; Stevens et al., 2016). As neurodynamic organizations regularly occur during silence, speech is an unlikely source for most organizations (Stevens and Galloway, 2014, 2015). Power spectral density (PSD) was estimated with the Welch method (Welch, 1967). Commercial EEG headsets with both dry and wet electrodes have been used from multiple vendors with the number of sensors ranging from five to nineteen. A greater number of electrodes allow more detailed analysis of the spatial locations of the sources of uncertainty in the brain.

### Team and Individual Neurodynamic Modeling

The modeling goal was to develop a multi-modal, multi-level system that would provide neurodynamic measures from each team member at a 1 Hz resolution that could be quantitatively compared across sensor sites (i.e., the occipital lobe vs. the motor cortex) and the 1–40 Hz frequency spectra from each person.

**Figure 2:**
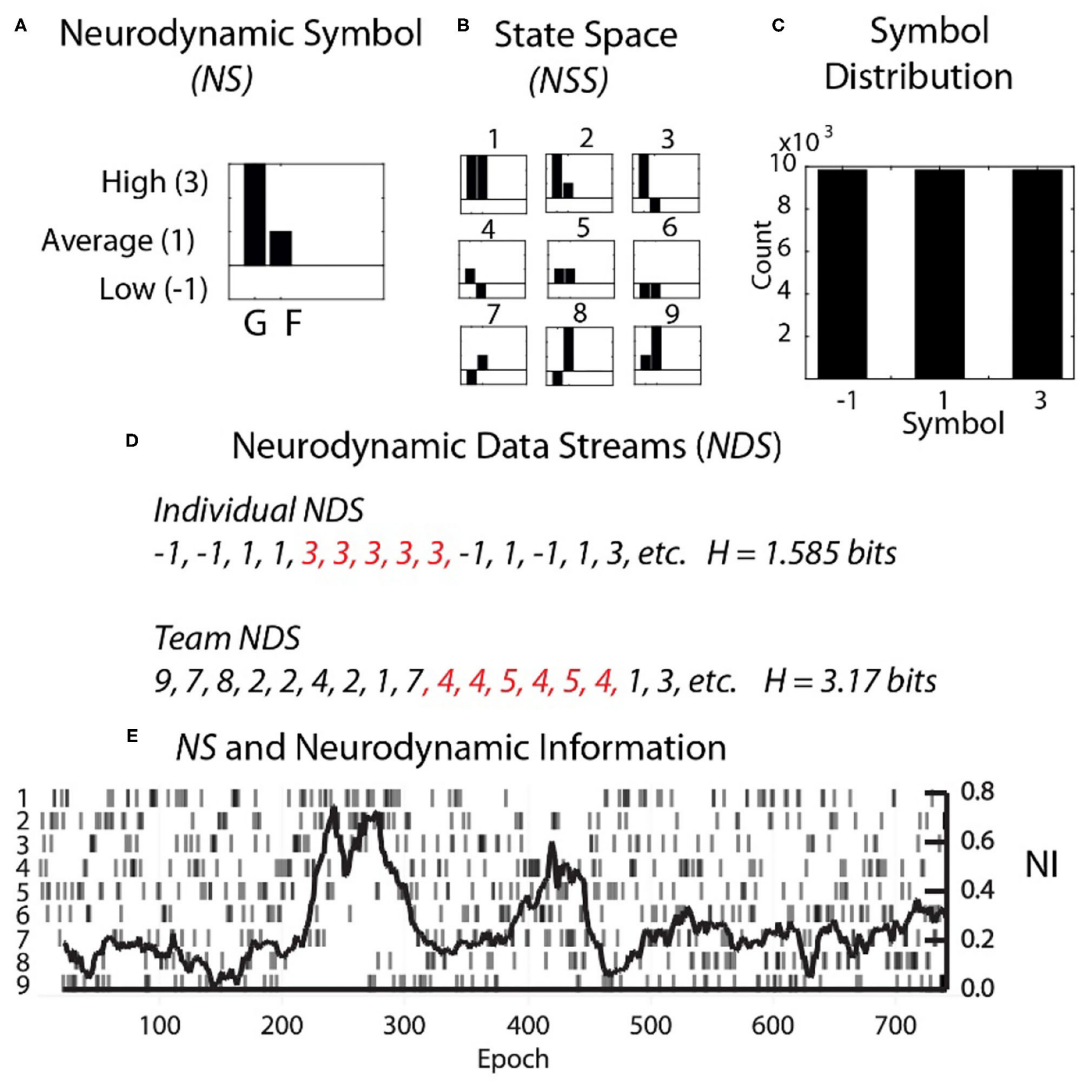
Team and individual neurodynamic modeling of a dyad. **(A)** A sample Neurodynamic Symbol (*NS)* showing a 1 s period where the EEG power was high for team member 1 (G), and average for team member 2 (F). **(B)** The nine-symbol Neurodynamic State Space (*NSS)* for two persons with three EEG power levels. **(C)** The distributions of the −1, 1, and 3 symbols need to be equal for accurate quantitative entropy comparisons. **(D)** Neurodynamic Data Streams (*NDS*) are symbol sequences that span the performance. For a dyad they are the team symbols in Figure 2B, while for individual team members they would be the−1, 1 and 3 values used symbolically. Note in Figure 2D that the symbol expression for both team and individual *NDS* were not random but punctuated by periods of symbol repeats which elevated the *NI*. Periods of symbol repeats were quantitated using entropy calculations over a 60 s window that was updated each second. **(E)** The NDS and the neurodynamic information tracing is shown for the dyad at the C3 sensor for the 18 Hz frequency.

Each second the EEG power spectral density *(PSD)* levels were separated into the performance averaged high, medium, and low power levels and assigned the symbols 3, 1, and −1. The entire performance of any team member could then be described by several hundred parallel data streams of −1, 1, or 3's (Figure 2). The data from each team member (shown for a dyad in Figure 2A) was then combined to create a composite neurodynamic symbol (*NS*) that represented the team state. With two persons and three states, the team symbolic history consisted of data streams of the symbols 1 to 9 and a maximum entropy of 3.17 bits (Figure 2B).

A single neurodynamic symbol contains no information; it has an entropy of 0. The detection and quantitation of uncertainty and hesitation used information modeling over the Neurodynamic Data Streams (*NDS*), followed by the calculation of the entropy (Shannon, 1948, 1951) of the symbol distributions that were modeled over 60 s that were updated each second.

The neurodynamic history of the team's performance described in Figure 2 was only a partial history based on a single 1 Hz frequency bin from a single EEG sensor. For a 19 sensor EEG montage, with each sensor containing forty 1 Hz bins, this process would be repeated 760 times.

**Figure 3:**
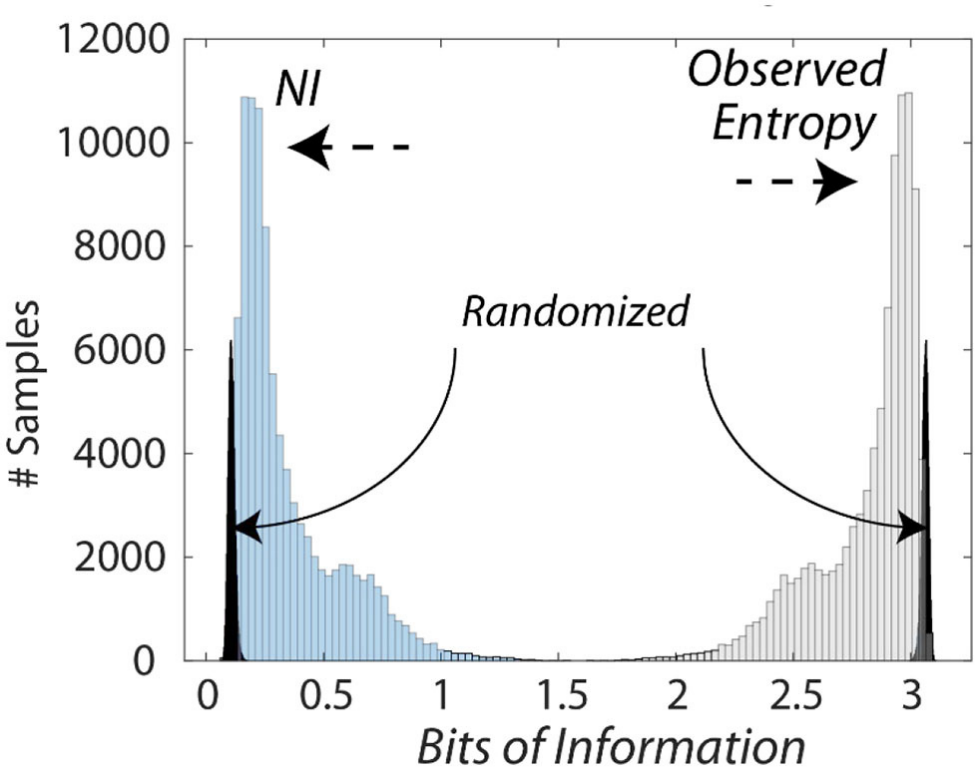
Histogram of the information levels in a medical flight team. This figure shows the relationship between the entropy and the *NI* measures of the medical flight team detailed in Figure 8. The average *NI* was 0.27 bits while the *NI* where the *NDS* were randomized prior to modeling was 0.09 bits which was significantly lower than the non-randomized data (*Z* = −5.5 *P* < 0.001), and removed the long-tailed distribution.

The bits of information calculated from the entropy measures were subtracted from the maximum entropy for the number of unique symbols to provide Neurodynamic Information (*NI*), a positive value of information. This relationship is shown in Figure 3 which compares the distributions of entropy and *NI* for the Medical Flight (Life Flight) trauma team performance described below. This figure also shows the changes in the distributions when the *NS* data streams were randomized six times before modeling.

### Bounding the Limits of Information

The entropy of the neurodynamic symbol streams can never be greater than the maximum of the number of symbols; for three PSD levels these would be 4.75 bits for a 27 symbol three person team, 3.17 bits for a nine symbol dyad, or 1.59 bits for an individual. The entropy can also never be lower than 0, which is the entropy of a single symbol. These mathematical limits mean that the entropy of any team of three persons where the EEG is separated into three levels will have entropy levels between 0 and 4.75 bits.

### Calculating the EEG Power Values

To preserve the activation-deactivation meanings of EEG the *NDS* were modeled numerically as well as symbolically using the −1, 1, and 3 values, which enabled dynamic comparisons between neurodynamic organizations expressed as *NI* bits and the underlying EEG power values (*EEG-PV*). A numeric moving average of 60 s was performed over *EEG-PV* data streams to align with the *NI*.

## Results

### Parsing the Neurodynamic Information of a Map Task Performance

**Figure 4:**
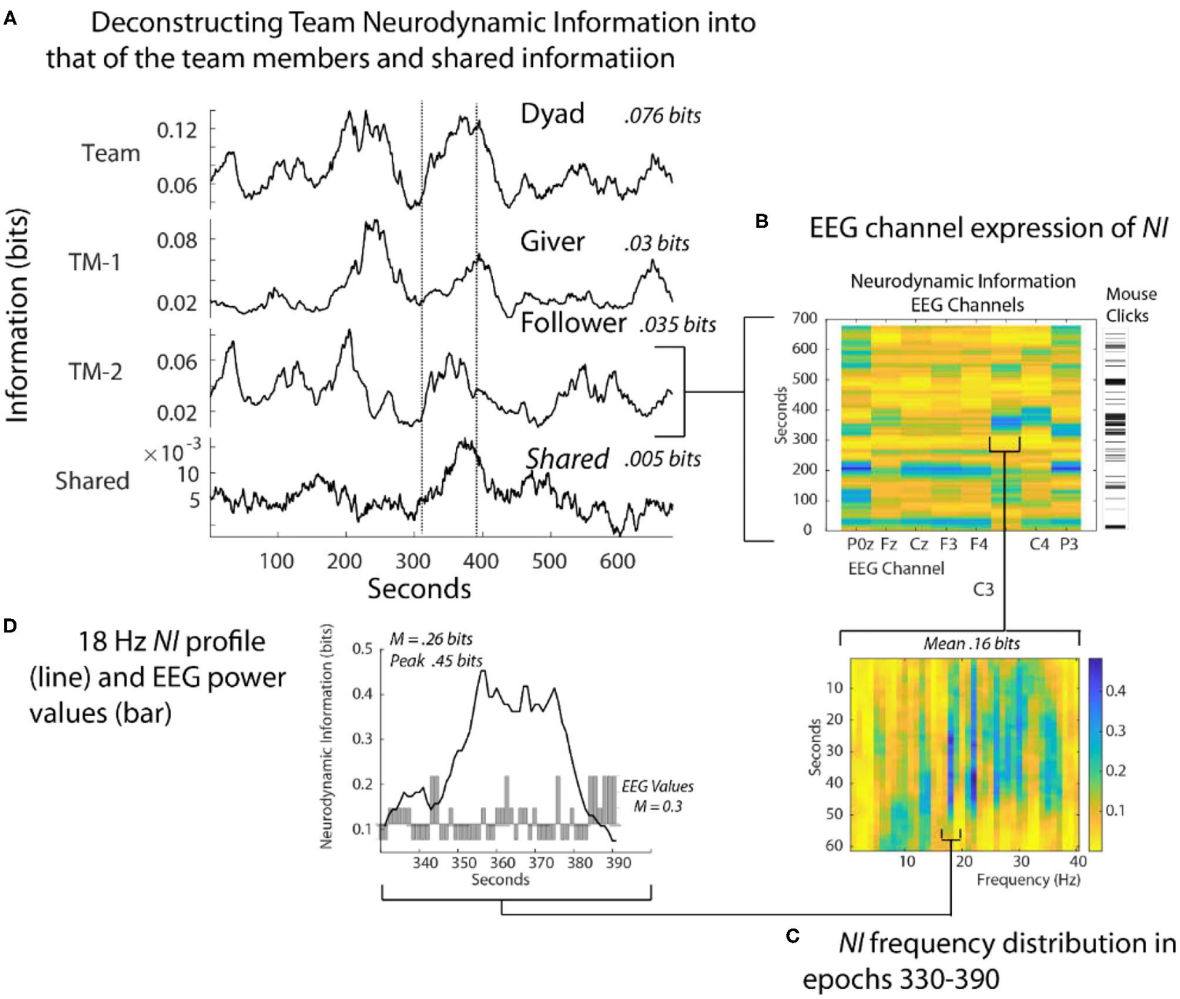
Quantitative comparisons of *NI* across spatial scales. **(A)** The bits of information of the team, team members, and their shared information is shown for a Map Task performance; the two lines are there for aligning the panels. **(B)** The *NI* dynamics at each sensor of the Follower. The bar to the right shows the density of his mouse clicks which increased around 330 s when he verbalized uncertainty. **(C)** The elevated *NI* at the C3 sensor is expanded for one segment (epoch 330-390 s) and displayed across the 1–40 Hz EEG frequency spectrum. **(D)** A profile plot of *NI* in the 18 Hz frequency band is overlaid with a bar plot of the −1, 1, and 3 *EEG-PV*. All *NI* values are shown after the subtraction of *NI* from parallel randomized *NDS*.

The dyad performed the Map Navigation Task (Doherty-Sneddon et al., 1997) with the Giver (G) verbally directing a second person, the Follower (F), in drawing a line through landmarks on (F's) computer screen (Stevens and Galloway, 2014, 2015, 2017). During the task, the team was exchanging information and (F) was drawing paths with the computer mouse around the task landmarks. Around 350 s (F) had difficulties controlling the cursor with the computer mouse and while attempting to gain control, began rapidly clicking it to send commands (Figure 4B). As the Follower became frustrated [indicated by (F) speech], the *NI* first increased in the parietal region (P0z, P3) and subsequently to the pre-motor/motor region (C3 and C4 sensors).

At a quantitative level, restricting the analysis to the EEG sensor level raised the average *NI* of the Follower from 0.035 bits to 0.16 bits. Restricting further to the 18-Hz frequency during the 60 s of peak activity of the C3 sensor further increased the average *NI* to 0.26 bits, reaching a peak fifteen times greater than the Follower's average *NI* in Figure 4A. This peak of 0.46 bits of *NI* was ~30% of that possible for three symbols (1.57 bits).

Lastly, a 60 s moving average of the EEG power values (*EEG-PV*) was calculated for the 18 Hz segment using the −1, 1, and 3 values numerically. The bar graph in the lower part of Figure 4D showed a mean power value of 0.26, which was significantly lower than the performance average (i.e., the mean performance level of equal numbers of −1, 1 and 3 values) of 1.0 (*Z*= –2.97*, p* < 0.05). These results suggest that the organization that produced the elevated *NI* resulted from persistent deactivation of mu rhythms, which occurred when (F) visualized and performed drawing movements (Caetano et al., 2007; Tognoli and Kelso, 2015).

### Transforming EEG μ-Volts Into Information Bits Creates New Behavior-Related Properties

**Figure 5:**
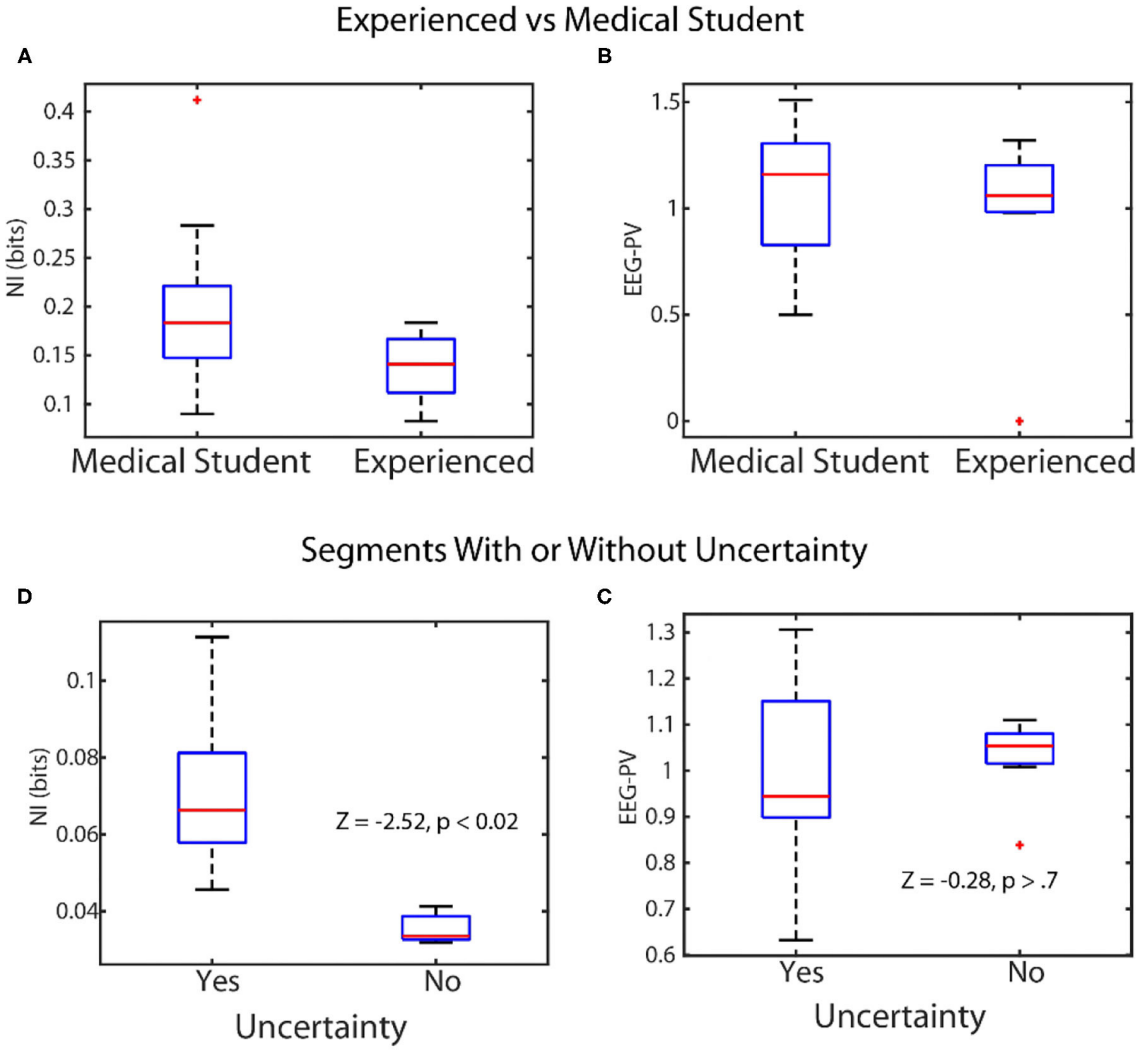
Linking levels of *NI* and *EEG-PV* with observable behaviors. Top: The scalp-averaged levels of *NI* **(A)**, and *EEG-PV* **(B)** were compared between fourth-year medical students (*n* = *15*) and experienced operating room physicians and nurses *(n* = *12*) while they performed simulations of patient ventilation. *Bottom:* The *NI* **(C)** and *EEG-PV* **(D)** were compared for segments from MT performances where uncertainty was (*n* = 29) or was not (*n* = 10) being expressed.

The across-scale parsing of neurodynamic information from the team to the micro level dynamics becomes interrupted at the level of EEG power where symbols representing either activated or de-activated EEG power resulted in elevated neurodynamic organization (Figure 4D). We therefore asked which was the better predictor of team and team member behavior: a measure of organization, (*NI*, bits of information), or a measure of EEG power levels *(EEG-PV)*. This was tested in two contexts: (1) Distinguishing experienced physician and operating room nurses performances from those of medical students during simulation training; and, (2) distinguishing performance segments associated with verbal uncertainty from those without (Figure 5).

In the first experiment four experienced and five medical student teams performed patient ventilation simulations and the scalp averaged *NI* and *EEG-PV* were modeled. The *NI* levels of medical students (Figure 5A) were significantly higher (median = 0.19 bits), than those of experienced practitioner (Figure 5B) (median = 0.12, *Z* = −2.0, *p* = 0.04, Wilcoxon). A similar comparison using *EEG-PV* from which the *NI* were derived showed no across-group differences; medical student (median = 1.16), experienced practitioners (median = 1.11, *Z* = −0.16, *p* = 0.87*)*.

In the second experiment we asked whether the *NI* and *EEG-PV* could equally distinguish MT performance segments where uncertainty was present or absent. The uncertainty was determined by verbalizations of (G) or (F). Twenty-nine segments from six MT performances were isolated where either (G) or (F) verbally indicated uncertainty (71 to 125 s in length (mean/SD = 96.1 ± 17.2 s). These utterances indicated not knowing what to do in the task, confusion about navigation instructions due to mirror map images of (G) and (F), discussions regarding the need to start over, or expressed difficulty during periods where the drawing tool was unresponsive. The *NI* (Figure 5C) and *EEG-PV* (Figure 5D) in these segments were compared with ten similarly sized segments where speech indicated no uncertainty or stress. The data for this experiment were acquired as part of a study where unsupervised artificial neural networks were used to categorize the neurodynamic uncertainty of Map Task dyad teams (Stevens and Galloway, 2019).

A Wilcoxon comparison of the *NI* bits was significantly different between segments with (median = 0.072), and without uncertainty (median = 0.033, *Z* = 2.52, *p* < 0.02), while a similar comparison using the *EEG-PV* was not different between the segments with (median = 0.96) or without expressed uncertainty (median = 1.05, *Z* = −0.28, *p* = 0.77).

This comparison confirmed for healthcare teams that the level of neurodynamic organization of experts was lower than that of novices. The data also indicated that the neurodynamic organizational properties of *NI* were more reflective of macro-scale behaviors than the underlying EEG power values from which the *NI* was derived. In other words, the data streams consisting of high, average and low *EEG-PV* lacked the information held by *NI* that enables linkages to be made with higher level behavioral measures of teamwork.

### Neurodynamics of Submarine Piloting and Navigation

The dynamical relationships between *NI* and *EEG-PV* were next studied with an expert submarine navigation team who were in port training for a new deployment. The goals were to determine: (1) whether discrete *NI* dynamics would be seen during a high stakes, high fidelity training exercise when the analysis was restricted to that of an individual sensor and EEG frequency; and, (2) whether the increased *NI* resulted from persistently activated or deactivated EEG power.

**Figure 6:**
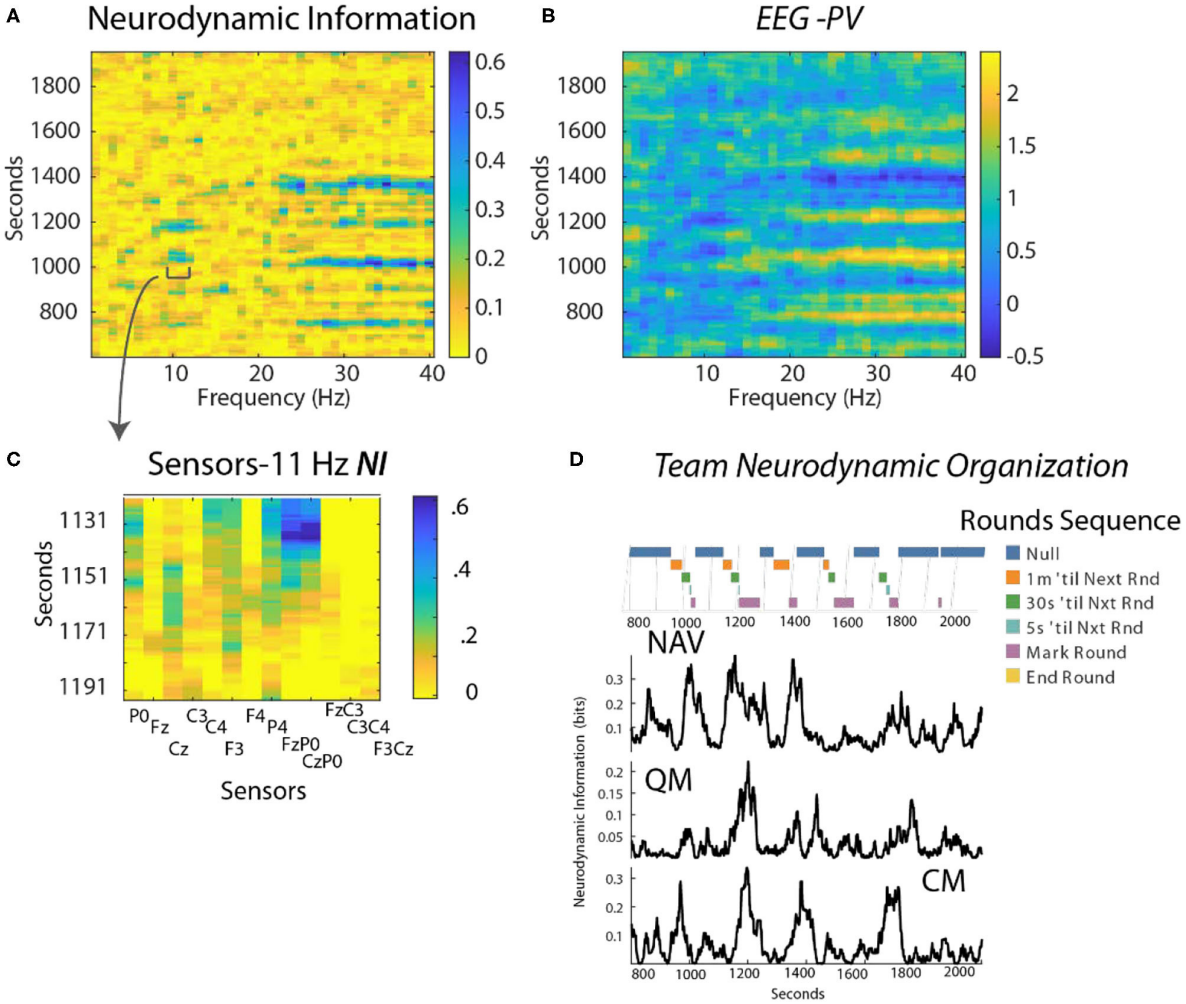
**(A)** Time x Frequency x *NI* plot. The color bar indicates the bits of *NI*. **(B)** Time x Frequency x *EEG-PV* plot. The color bar shows the level of *EEG-PV* calculated from the −1, 1, and 3 values; the average value for each frequency band was 1.0. **(C)** The data were modeled from the FzP0 dipole of the CM which had the highest *NI* levels of the channels. **(D)** The countdowns for the last minute of the five Rounds sequences are plotted vs. time. These figures plot the *NI* for the Navigator (NV mean *NI* = 0.23 bits, randomized = 0.01) Quartermaster (QM mean NI = 0.14 bits, randomized = 0.017) and the Contact Manager (CM, mean *NI* = 0.12 bits, randomized = 0.03).

This analysis focused on a periodic training activity called “Rounds” where the position of the submarine is estimated every 3 min using visual and electronic aids. Preliminary studies identified the FzP0 dipole as having the greatest *NI* in the 10–11 Hz region during periods of Rounds and this is shown in Figure 6C for the Contact Manager (CM).

Time-frequency plots showed *NI* elevations in the 10–11 Hz, and the 23–40 Hz regions that were spaced at ~3 min. intervals (Figure 6A shown for the CM). The *EEG-PV* profiles (Figure 6B) showed segments, particularly in the 23–40 Hz bands, with either activated (light bands) or deactivated EEG power (dark bands) that visually aligned near the periods of increased *NI*.

The *NI* were analyzed for the NV, QM and CM who were the primary control room navigation team (Figure 6D). The 11 Hz activity is highlighted as it was a frequency distant from the broader 23–40 Hz activity. It was also more discrete, thus allowing a better estimation of the duration of each peak. The individual dynamics are aligned with a timeline marking the 1 min countdown events of the Rounds sequence. The *NI* peaks for the three team members were weakly correlated (NV-QM, *r* = 0.36; NV-CM, *r* = 0.3; QM-CM, r = 0.34; *p* < 0.01 for all), but not synchronous.

**Figure 7:**
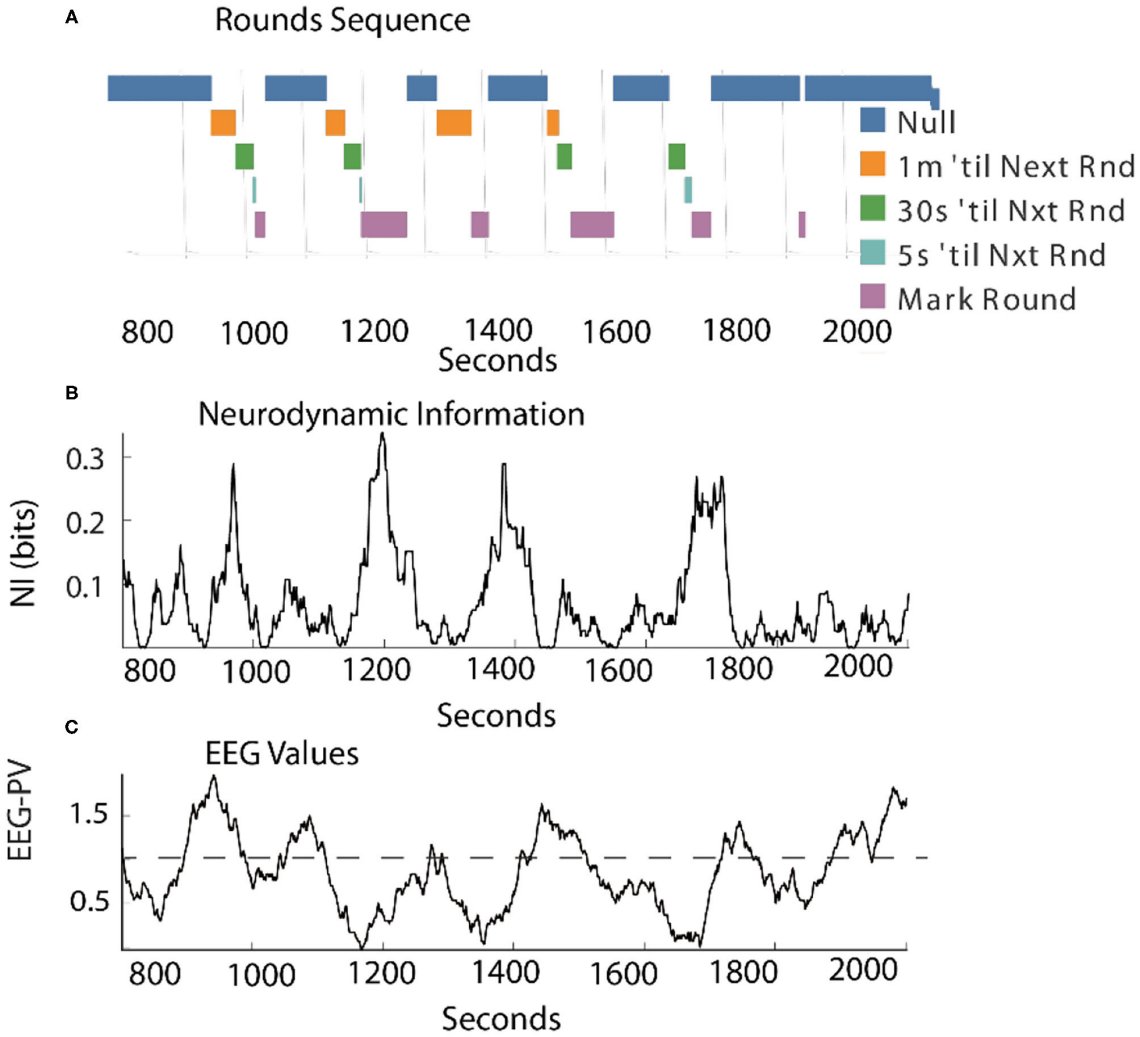
Comparison of the *NI* and *EEG-PV* dynamics of the Contact Manager. **(A)** The countdowns for the last minute of the five Rounds sequences are plotted vs. time. These figures plot the *NI* **(B)** and the *EEG-PV* **(C)** for the CM. The dotted line in **(C)** shows the 11 Hz frequency of 1.0 that demarcates positive and negative values. All values are from the FzP0 dipole of each person. The average value of the randomized *NI* was 0.01 bits. The correlation between **(B)** and **(C)** was *r* = –0.41.

As shown in Figure 7, when measured continuously the correlation between levels of *NI* and *EEG-PV* was moderately negative (*r* = −0.41).

### Parsing the Neurodynamic Information of a Medical Flight Team

**Figure 8:**
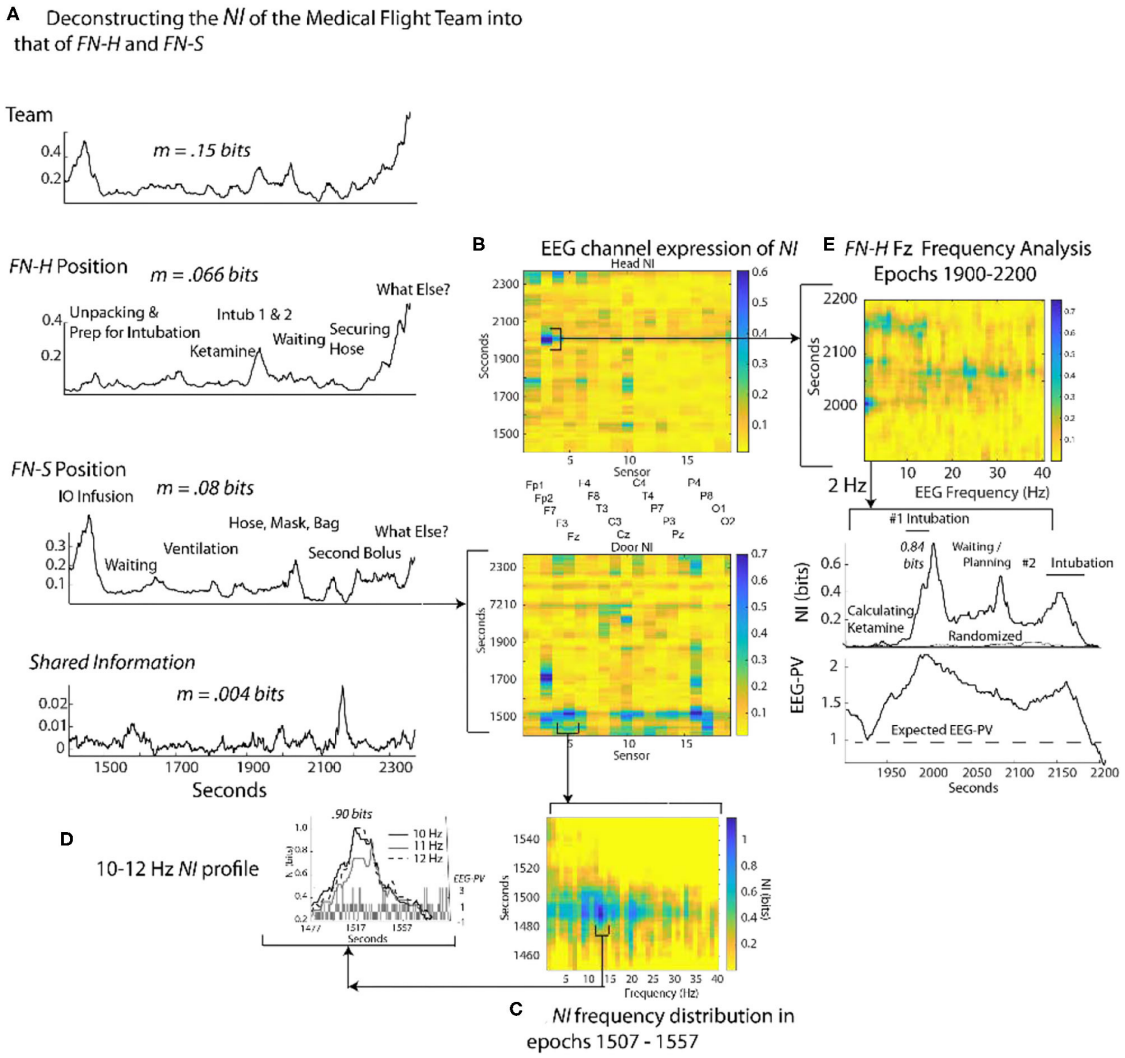
Quantitative comparison of *NI* levels for a medical flight team. **(A)** The bits of information of the team, team members and the member's shared information. **(B)** The *NI* dynamics at each sensor at for the head (*FN-H*) and side (*FN-S*) positions. **(C)** The elevated *NI* at the Fz sensor is expanded for one segment (epoch 1507–1557 s) of the *FN-S* and displayed across the 1–40 Hz EEG frequency spectrum. **(D)** A profile plot of *NI* in the 10–12 Hz frequency bands is shown with a bar chart of *EEG-PV* underneath. **(E)** A drill-down sequence for *FN-H* at the sensor level, followed by isolation of the 2 Hz *NI* and *EEG-PV* for a segment (1,900–2,200 s) of the P7 channel. The correlation between *NI* and *EEG-PV* was *r* = 0.84.

To gauge the generality of the framework in Figure 3, a similar analysis was performed with an experienced two-person medical flight team where EEG was acquired within a stationary helicopter environment. The task was simulated pediatric bronchiolitis. The Flight Nurse at the head of the child (*FN-H*) began by unpacking the supplies needed for establishing an airway for the infant (Figure 8A). (*FN-H*) then calculated and diluted a dosage of ketamine hydrochloride (because of its bronchodilatory properties) for sedation in order to perform the ventilation procedure (i.e., intubation). Two intubation attempts were performed followed by a period of infant monitoring; the elevated *NI* occurred during the intubation procedures.

The Flight Nurse at the side position (*FN-S*) began with an intraosseous infusion (IO) that required drilling into the child's tibia. Next *FN-S* rapidly provided rescue ventilation with bag-valve-mask ventilation prior to and after the intubations. For *FN-S*, the periods of elevated *NI* occurred during the IO infusion.

The average *NI* of the two team members (*FN-H* = 0.066 bits; *FN-S* = 0.08 bits) and the shared information equaled the information of the team (0.15 bits), a relationship similar to that seen in Figure 4.

At the sensor level the periods of elevated *NI* involved a subset of EEG channels, which for *FN-H* were those in the frontal region, while for *FN-S* they were mostly of the central and parietal regions (Figure 8B). The peaks in the most active EEG channels were often discrete and the *NI* was enriched over five times the session average (Figure 8C).

Similar to Figure 4, sample *NI* peaks were further analyzed for the events when *FN-S* was inserting a needle into the tibia for fluid infusions (Figures 8A,C,D). The *NI* peak highlighted in Figure 8C (the Fz channel) of *FN-S* was localized to the 10-12 Hz frequency range and was symmetrical with a half prominence of 36 s (Figure 8D). This peak had *NI* level (0.9 bits) which was greater than half of the theoretical maximum of 1.59 bits. The bar chart in the lower level of Figure 8D showed a mean power level of 0.2 which was lower than the session average of 1.0 indicating EEG deactivation during this period.

The *NI* peak of *FN-H* highlighted in Figure 8B (the F7 channel) was predominantly in the 2–4 Hz frequencies (i.e., delta wave) and was highest (0.84 bits) during the failed intubation attempt (Figure 8E). During the gap between the two intubations there were additional *NI* elevations in the beta and low gamma range.

### Estimating the Magnitude and Duration of *NI* Peaks

Scalp-averaged *NI* levels vary based on their frequency, magnitudes and durations of neurodynamic organization. From a training and feedback perspective important questions are: How frequently does uncertainty occur? What is the level of uncertainty? How long is the uncertainty likely to last? The frequency and magnitude of *NI* peaks can be estimated by peak-finding routines that identify peaks based on the magnitude and the relationships with their neighbors.

Using these routines, the incidence of *NI* peaks was estimated for the team members of three Life Flight teams each second. For maximum resolution, this was calculated each second for each of the 1–40 Hz bins for each of the 19 sensors (Table 1). Across the six team members the peaks of elevated *NI* represented 7–8 percent of the total performance times.

**Table 1:** Incidence of periods of uncertainty at the EEG frequency level.

		**Total epochs**	**Epochs with peak *NI***	**Percent**
LF1S1	*FN-H*	737,960	56,590	0.08
	*FN-S*	737,960	54,969	0.07
LF2S1	*FN-H*	1,357,360	95,407	0.07
	*FN-S*	1,357,360	98,503	0.07
LF3S1	*FN-H*	1,812,600	137,707	0.08
	*FN-S*	1,812,600	132,916	0.07

*The uncertainty peaks are defined as ones greater than its two neighbors and had a prominence of at least 0.005 bits*.

**Figure 9:**
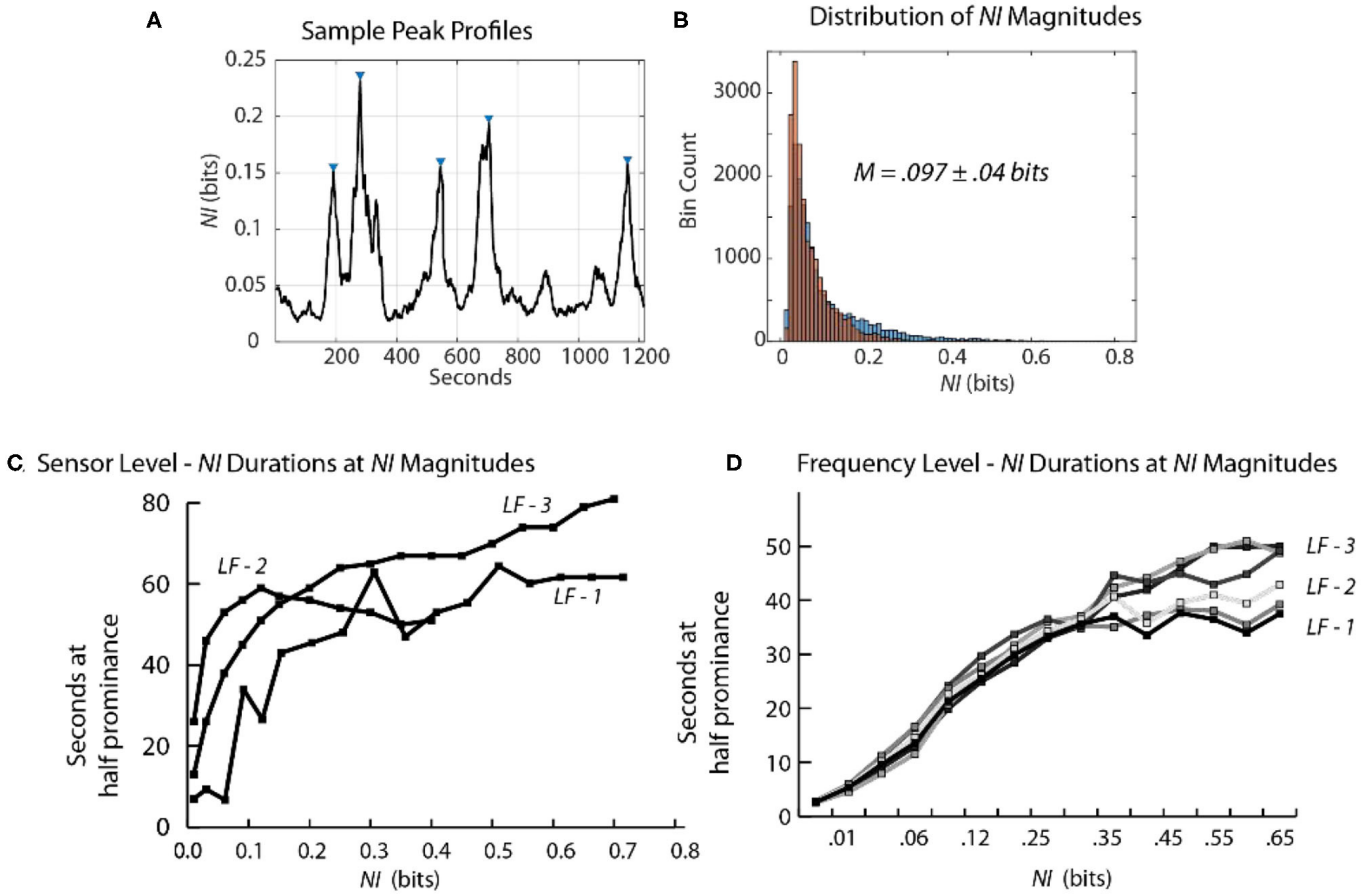
Relationship between the magnitude and duration of *NI* peaks. **(A)** A segment of a Life Flight team *NDS* showing peak identifications, using the *findpeaks.m* MATLAB® function. The peak magnitudes resulting from this function are based on having a minimum level of magnitude, which was 0.1 bits for this plot. **(B)** The second-by-second *NI* distributions from the 19 sensors of the *FN-H* and *FN-S* of LF2. **(C)** This plot shows the duration of peaks (y-axis) within different magnitude intervals (x-axis). **(D)** The duration of peaks is shown for the six team members when the data streams included the 1–40 Hz frequency bands of each sensor.

The *NI* profiles in sections Parsing the Neurodynamic Information of a Map Task Performance, Neurodynamics of Submarine Piloting and Navigation, and Parsing the Neurodynamic Information of a Medical Flight Team showed that as the analyses proceeded from the team toward the EEG frequency level the *NI* peaks became more discrete (Figure 9A) increasing the reliability of the magnitude and duration estimates.

At the sensor level, when estimated with a minimum peak prominence of 0.1 bits the mean peak duration was 41 ± 18 s while the mean *NI* magnitude was 0.097 ± 0.04 bits (Figure 9B). When the *NI* peak duration was estimated at different prominence levels, it rapidly increased for the first 0.1–0.15 bits and then began to plateau with a duration ~55–60 s.

When similar estimates were performed at the frequency level a similar plateau was observed beginning around 0.2 bits. The plateau was lower (30–50 s) suggesting further peak resolution when parsing to this level (Figures 9C,D).

### Performance-Wide *NI* and *EEG-PV* Interactions Are Complex

The parsing of NI across different spatial and temporal scales in Figures 4, 7, and 8 provided evidence for both positive and negative correlations between *NI* and *EEG-PV* when examined at the level of individual sensors and frequencies.

**Figure 10:**
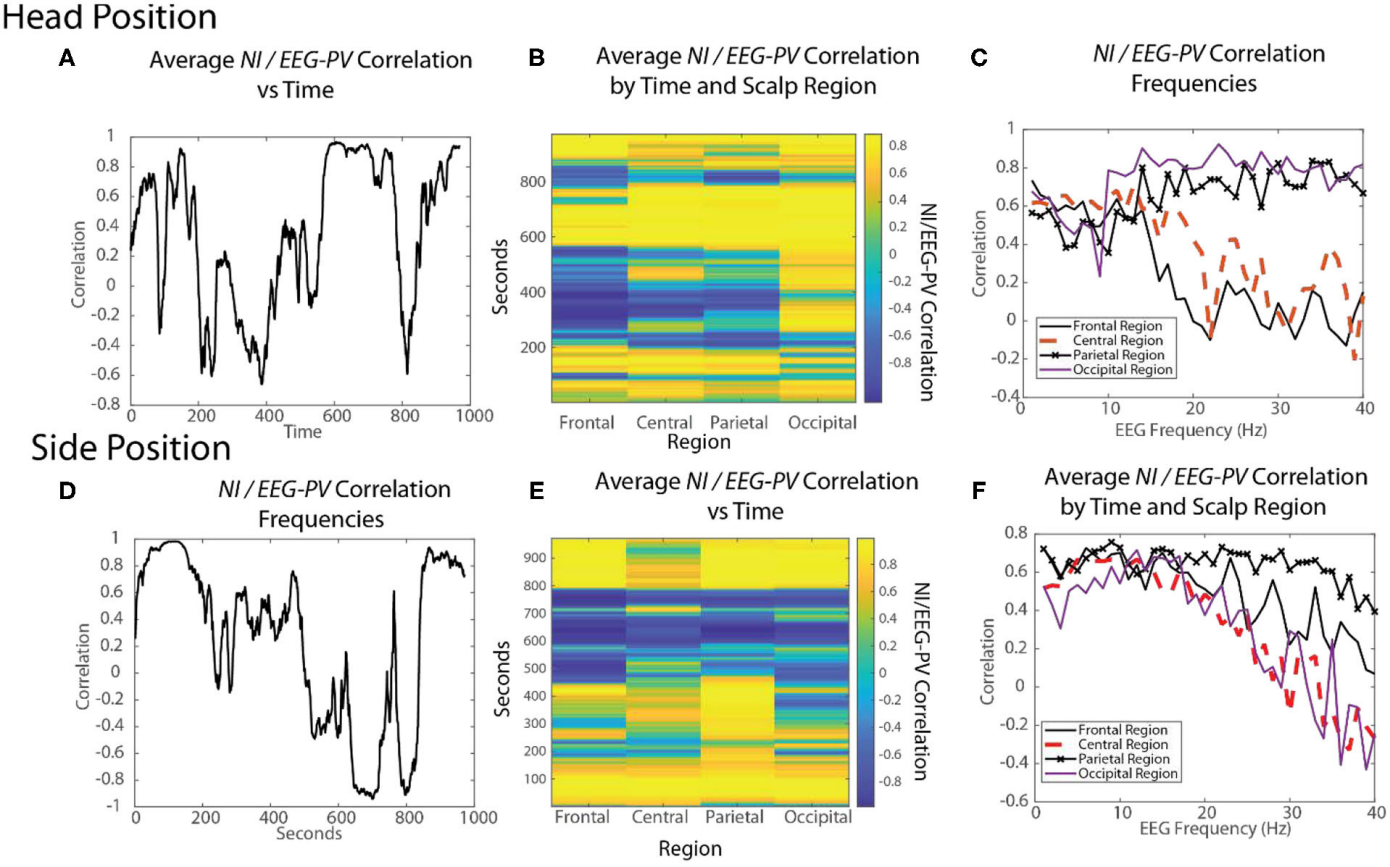
The changing relationships of *NI* and *EEG-PV* during an ecologically valid task. The correlation between parallel *NI* and *EEG-PV* data streams were measured for *FN-H* (top) and *FN-S* during a Life flight performance (LF1). The correlation data was aggregated across the scalp **(A,D)**, regions of the scalp **(B,E)** or at the EEG frequency level for regions of the scalp **(C,F)**.

For *FN-H* the scalp-averaged correlations (Figure 10A) fluctuated between *r* > 0.9 to *r* < −0.65 over the performance. The greatest positive correlation (between 450 and 750 s) coincided with the two endotracheal intubation attempts where there was both high *NI* and high *EEG-PV*. The periods of negative correlation were those where *FN-H* was preparing supplies or monitoring vital signs before and after the change of intubation techniques.

For *FN-S* the scalp-averaged correlations for *FN-S* (Figure 10D) fluctuated between *r* > 0.9 to *r* < −0.9 with the greatest correlation at the beginning of the task while preparing the IO fluid port and the lowest while *FN-S* was watching *FN-H* attempt the two intubations.

The scalp-wide analysis was then segmented into the sensors in the frontal (Fp1, Fp2, F7, F3, Fz, F4 F8), central (T3, C3, Cz, C4, T4), parietal (P7, P3, Pz, P4, P8) and occipital (O1, O2) scalp regions. The correlations were then repeated *FN-H* (Figure 10B) and *FN-S* (Figure 10E) over a window of 60 s that was updated each second using data from these regions.

For *FN-H* the strongly positive *NI/EEG-PV* correlations were scalp-wide indicating a global involvement of all brain regions. The periods of negative correlations appeared more regionally local with the greatest concentration in the frontal region, and the least in the occipital region.

For *FN-S* the positive correlations at the beginning of the simulation were also scalp-wide, while the negative correlations were both locally and globally distributed (Figure 10E).

The final perspective of the *NI* / *EEG-PV* correlations was across the EEG frequency spectrum for the different scalp regions (Figures 10C,F). For the *NDS*, from both *FN-H* and *FN-S*, the positive correlations were present over the 1–15 Hz frequencies. For *FN-H*, the correlations for the frontal and central regions became negative in the beta and gamma regions approaching *r* = −2, while those for the parietal and occipital regions remained high. This divergence in *NI/EEG-PV* correlations between low and high frequencies also occurred with the data from *FN-S*, but only the correlation in the parietal region remained high in the beta and gamma frequencies.

The high vs. low EEG power and *NI* relationships are therefore complex. There are suggestions of global brain involvement with higher EEG power which preferentially occurs in the 1–15 Hz EEG bands. As the *NI* became more regionally local, the correlation with *EEG-PV* became more neutral to negative implying increased neurodynamic organization as a result of temporally persistent low *EEG-PV*.

## Discussion

Investigators are increasingly turning to sensor technologies, including EEG, to better understand and optimize team training and support, but with little theory to guide them in how to best represent data across the heterogeneous neuronal timescales at micro levels (Murray et al., 2014) and the heterogeneous timescales of teamwork at the macro levels. One challenge is finding representations that bridge the gap between implicit neural and physiologic variables that are inaccessible to visual inspection and understanding (micro scale), with those that are. A second challenge is moving the technologies toward real-time continuous data collection and analysis, which for neural data will almost certainly require abstractions of the lowest levels of neural activity.

The first was that the EEG data streams that were modeled at a 1 Hz resolution contained periods of neurodynamic organization lasting seconds to minutes that under closer inspection linked with observable behaviors like the feeling of uncertainty, or as yet unknown qualities that distinguish experienced operating room staff from medical students. A second theme was that team neurodynamic data when parsed to the level of individual channels and frequencies showed discrete *NI* peaks of varying frequency, magnitude, and duration. A third theme was that both activated and suppressed EEG amplitudes contributed to elevated *NI*.

The results in section Transforming EEG μ-volts into information bits creates new behavior-related properties parallel what we previously saw when comparing *NI* levels and instructor ratings of submarine navigation team performance where there was an inverse correlation between levels of *NI* and team ratings (Stevens et al., 2017). Combined, the two datasets indicate that higher performing teams are generally less neurodynamically organized than lower performing teams.

The inverse correlation between levels of *NI* and the experience also has parallels with the idea of neural efficiency in that they are both indicators of more efficient energy states. Neural efficiency refers to the patterns of reduced brain activity with equal or superior performance and was originally described as it related to intelligence (Neubauer and Fink, 2009). The idea of neural efficiency has recently been expanded to include differences between skilled basketball and volleyball players. Studies by Zhang et al. (2019) showed that when imagining performing skills in the domain of their expertise the players showed greater neural efficiency (as measured by fMRI) than when imagining performing in the other sport.

The higher *NI* levels in less experienced/proficient individuals may be partially explained by the finding that periods of *NI* increases often occur when there is uncertainty. Uncertainty arises when results do not match expectations, and from the ideas of neural efficiency these might be periods where the complexities of the task outweighed the developed expertise, Less experienced individuals are more likely to experience increases in the frequency, magnitude and/or duration of uncertainty, and the ability to quantitatively measure these dimensions of uncertainty would be useful for probing the details of the novice-expert continuum.

The frequency of encountering uncertain events is a factor of experience (Kennedy et al., 2005; Stevens et al., 2017, 2018). The better prepared an individual/team member is, through training and practice, the less likely s/he is to encounter a surprising event. In part this will be due to the possession of a larger repertoire of complied episodes. One of the consequences of extensive training is that many of the possible perturbations to the team will have been experienced (i.e., this uncertainty would be expected as opposed to unexpected) and the (low) likelihood of their occurrence will have been factored in Soltani and Izquierdo (2019).

The magnitude of uncertainty reflects the costs of searching across levels of cognition to resolve it (Zénon et al., 2018). Events like the loss of mouse cursor control in Figure 4, cause the team to abandon a search of the library of episodes hoping for a relevant match, and instead adopt an exploratory strategy. Such exploration would no longer be spatially and temporally local within the brain and could involve prolonged and distributed iterations of the predictive coding – action cycles of the information hierarchy while evidence is triangulated (Clark, 2016; Shipp, 2016; Yon et al., 2020). During predictive coding, each level of the hierarchy predicts representations in the level below, via backward connections. Predictions are fed backward in the hierarchy and reciprocated by prediction error in the forward direction, acting to modify the representation of the outside world at increasing levels of abstraction, and optimize the nature of perception over a series of iterations. An example would be again from the Map Task in Figure 4 where the highlighted segment is one of a sequence involving elevated *NI* being detected first in the parietal region (P3) and then moving to the motor regions (C3 and C4 sensors) while options for controlling the mouse are explored.

The factors affecting the third dynamical component, duration, are less understood. Determining how long hesitation will last may be the most difficult to predict, yet probably the most valuable for training. To estimate the duration of uncertainty in these expert teams we parsed the *NI* data streams into smaller spatial and temporal scales. The data shown in sections Parsing the Neurodynamic Information of a Map Task Performance, Neurodynamics of Submarine Piloting and Navigation, and Parsing the Neurodynamic Information of a Medical Flight Team illustrate how the structural dynamics of *NI* became more discrete as the neurodynamic analysis of the team was extended to the individual, sensor and then the EEG frequency level. These discrete peaks were also enriched in the levels of *NI* with extended peaks (40–60 s) approaching the upper theoretical limits of the entropy of the system.

The regional and frequency localization of these peaks, and their association with periods of stress or uncertainty suggests that the responses of experts to these (most likely expected) events are regionally local in the brain. Examples included in this paper are the deactivation of mu rhythms in Figure 4, the activation of delta rhythms in Figure 8 and the (mostly) deactivated alpha rhythms in Figure 7.

The elevated neurodynamic organization of delta waves (2–4 Hz) during periods of task difficulty and of greatest *NI* levels was unexpected. In the Life Flight performance illustrated in Figure 8 the two team members showed elevated *NI* in the 1–4 Hz region during two delicate motor activities both of which require focused attention and precise movements. The elevated *NI* was greatest in the frontal scalp sensors but was detectable scalp-wide. This global scalp distribution is consistent with the general idea in neuroscience that low frequencies tend to be global expressed, while the higher frequencies tend to be more local (Buzaki, 2006).

Delta waves are widely used as proxy for a sleep homeostatic process and have primarily been reported in the context of sleep-wake driven changes (Hubbard et al., 2020), but more recently they have gained attention as a modulator of executive action of motor function (Harmony, 2013).

Recent studies of delta wave suppression have taken advantage of the physiological mirror response where motor activity involuntarily occurs in the opposite side of the body when deliberate motor activity like hand gesturing are planned (Maudrich et al., 2020). When participants were made aware of this activity and asked to inhibit any involuntary co-activation their directed attention exerted an inhibitory drive on the involuntary motor output, and this suppression was accompanied by increased delta power in frontal areas. An important aspect of these studies was that the participants were able to suppress this motor response without any special training or online feedback, just by becoming aware that the mirroring response was occurring. This represents one of the simplest forms of neurofeedback.

The finding of elevated delta *NI* and *EEG-PV* is unusual, but not unique to Life Flight simulations. Recently we studied the neurodynamics of a neurosurgery team operating to decompress the peroneal nerve of a live patient (Stevens et al., 2019). For the final clipping of the offending tendon the primary neurosurgeon relieved the resident-neurosurgeon. During the remaining seven minutes elevated *NI* in the delta region was the dominant neurodynamic feature. The finding of elevated delta *NI* in the neurosurgeon and expert Life Flight team members may indicate that one of the characteristics of expertise is the finer control of muscle movements during task-critical areas where they have had extensive training.

The analyzing at the EEG channel and frequency levels also enabled estimates to be made of the duration of elevated *NI*. At the sensor level, the duration of *NI* peaks began to plateau around 55–65 s when a *NI* magnitude of ~0.2 bits was reached. When the analysis was repeated using data sampled at the frequency level a plateau was reached around 0.25 bits with a duration of 35–45 s. These results indicate that deriving quantitative estimates of the frequency, magnitude and duration of uncertainty can be realistically accomplished.

The final theme was the relationship between *NI* levels and levels of activated vs. inactivated EEG amplitude which was clearly complex. The results establish that elevated *NI* levels can result from both periods of EEG activation as well as deactivation. The associations with macro-scale behaviors however were greater for *NI* than for *EEG-PV* implying that the EEG power/organization junction is important for passing messages from micro-scale to macro-scales of teamwork.

The presence of an organizational system of information that parallels the amplitude of EEG rhythms is important as it provides a greatly reduced data dimension while retaining the essential system features, i.e., linkages to higher scale behaviors. In this way the combinatorial explosion of EEG rhythmic variables at micro levels become compressed into an intermediate system of information and organization which links to macro-scale team and team member behaviors.

## Data Availability Statement

The raw data supporting the conclusions of this article will be made available by the authors, without undue reservation.

## Ethics Statement

The studies involving human participants were reviewed and approved by Biomed IRB, San Diego CA, Naval Submarine Medical Research Board Order of Saint Francis Hospital Institutional Review Board. The patients/participants provided their written informed consent to participate in this study.

## Author Contributions

RS and TG co-designed the study and wrote the article. Both authors contributed to the article and approved the submitted version.

## Conflict of Interest

RS received his PhD in Molecular Genetics from Harvard University and is Professor (Emeritus), UCLA School of Medicine and a member of the UCLA Brain Research Institute. RS was also employed by The Learning Chameleon, Inc., a for profit educational technology corporation. His recent research had focused on using EEG-derived measures to investigate team neurodynamics in complex, real-world training settings. These studies were leading to quantitative teamwork models showing how teams dynamically reorganize in response to changing environments. TG was employed by, and was the director of cognitive electrophysiology research for The Learning Chameleon laboratory. She received her CPFDA, EFDA and CDA from Oregon Health and Sciences University in 1995 later specializing in several areas of process analysis. Her research interest blend the population based advantages of probabilistic performance modeling with the detection of neurophysiologic signals to help personalize and accelerate the individual and team learning processes.

## References

[B1] Beck J. M. Ma W. J. Kiani R. Hanks T. Churchland A. K. Roitman J.. (2008). Probabilistic population codes for Bayesian decision making. Neuron 60, 1142–1152. 10.1016/j.neuron.2008.09.021 19109917 PMC2742921

[B2] Bigdely-Shamlo N. Mullen T. Kothe C. Su K. M. Robbins K. A. (2015). The PREP pipeline: standardized preprocessing for large-scale EEG analysis. Front. Neuroinform. 9:B153. 10.3389/fninf.2015.00016 26150785 PMC4471356

[B3] Bodala I. P. Li J. Thaor M. V. Al-Nashas H. (2018). EEG and eye tracking demonstrate vigilance enhancement with challenge integration. Front. Hum. Neurosci. 10:273. 10.3389/fnhum.2016.00273 27375464 PMC4894919

[B4] Bonnefond M. Jensen O. (2015). Gamma activity coupled to alpha phase as a mechanism for top-down controlled gating. PLoS ONE 10:e012866. 10.1371/journal.pone.0128667 26039691 PMC4454652

[B5] Buzaki G. (2006). Rhythms of the Brain. New York, NY: Oxford University Press.

[B6] Caetano G. Jousmaki V. Hari R. (2007). Actor's and observers' primary motor cortices stabilize similarly after seen or heard motor actions. Proc. Natl. Acad. Sci. U.S.A. 104, 9058–9062. 10.1073/pnas.0702453104 17470782 PMC1859994

[B7] Clark A. (2016). Surfing Uncertainty: Prediction, Action and the Embodied Mind. New York, NY: Oxford University Press 10.1093/acprof:oso/9780190217013.001.0001

[B8] Cooper R. Shallice T. (2000). Contention scheduling and the control of routine activities. Cogn. Neuropsychol. 17, 297–338. 10.1080/026432900380427 20945185

[B9] Daniels B. Flack J. Krakauer D. (2017). Dual coding theory explains biphasic collective computation in neural decision-making. Front. Neurosci. 11:313. 10.3389/fnins.2017.00313 28634436 PMC5459926

[B10] Daw N. D. O'Doherty J. P. Dayan P. Seymour B. Dolan R. J. (2006). Cortical substrates for exploratory decisions in humans. Nature 441, 876–879. 10.1038/nature04766 16778890 PMC2635947

[B11] Delorme A. Makeig S. (2004). EEGLAB: an open source toolbox for analysis of single-trial EEG dynamics including independent component analysis. J. Neurosci. Methods 134, 9–21. 10.1016/j.jneumeth.2003.10.009 15102499

[B12] Delorme A. Sejnowski T. Makeig S. (2007). Enhanced detection of artifacts in EEG data using higher-order statistics and independent component analysis. Neuroimage 34, 1443–1449. 10.1016/j.neuroimage.2006.11.004 17188898 PMC2895624

[B13] Doherty-Sneddon G. Anderson A. O'Malley C. Langton S. Garrod S. Bruce V. (1997). Face-to-face and video-mediated communication: a comparison of dialogue structure and task performance. J. Exp. Psychol. Appl. 3, 105–125. 10.1037/1076-898X.3.2.105

[B14] Domenech P. Rheims S. Koechlin E. (2020). Neural mechanisms resolving exploitation-exploration dilemmas in the medial prefrontal cortex. Science 369:1076. 10.1126/science.abb0184 32855307

[B15] Dragomir E. Stih V. Portugues R. (2020). Evidence accumulation during a sensorimotor decision task revealed by whole-brain imaging. Nat. Neurosci. 23, 85–93. 10.1038/s41593-019-0535-8 31792463

[B16] Dunst B. Benedek M. Jauk E. Bergner S. Koschutnig K. Sommer M.. (2014). Neural efficiency as a function of task demands. Intelligence 42, 22–30. 10.1016/j.intell.2013.09.005 24489416 PMC3907682

[B17] Farooqui A. Manly T. (2018). Hierarchical cognition causes task related deactivations but not just in the default mode regions. eNeuro 5:ENEURO.0008-18.2018. 10.1101/297259 30627658 PMC6325562

[B18] Farooqui A. Manly T. (2019). We do as we construe: extended behavior construed as one task is executed as one cognitive activity. Psychol. Res. 83, 84–103. 10.1007/s00426-018-1051-2 30022243 PMC6373351

[B19] Flack J. (2017a). Life's Information Hierarchy. From Matter to Life: Information and Causality. New York, NY: Cambridge University Press.

[B20] Flack J. (2017b). Coarse-graining as a downward causation mechanism. Phil. Trans. R. Soc. A. 375:20160338. 10.1098/rsta.2016.0338 29133440 PMC5686398

[B21] Flack J. Erwin D. Elliot T. Krakauer D. (2012). “Timescales, symmetry, and uncertainty reduction in the origins of hierarchy in biological systems,” in Cooperation and its Evolution, eds Sterelny K. Joyce R. Calcott B. Fraser B., (Cambridge, MA: MIT press), 45–74.

[B22] Fox M. D. Snyder A. Z. Vincent J. L. Corbetta M. Van Essen D. C. Raichle M. E. (2005). The human brain is intrinsically organized into dynamic, anticorrelated functional networks. Proc. Natl. Acad. Sci. U.S.A. 102, 9673–9678. 10.1073/pnas.0504136102 15976020 PMC1157105

[B23] Friston K. (2010). The free-energy principle: a unified brain theory? Nat. Rev. Neurosci. 11, 127–138. 10.1038/nrn2787 20068583

[B24] Friston K. Kilner J. Harrison L. (2006). A free energy principle for the brain. J. Physiol. 100, 70–87. 17097864 10.1016/j.jphysparis.2006.10.001

[B25] Gorman J. Martin M. Dunbar T. Stevens R. Galloway T. Amazeen Linkens A. (2015). Cross-level effects between neurophysiology and communication during team training. Hum. Factors 58, 181–199. 10.1177/0018720815602575 26391663

[B26] Gusnard D. Raichle M. (2001). Searching for a baseline: functional imaging and the resting human brain. Nat. Rev. Neurosci. 2, 685–694. 10.1038/35094500 11584306

[B27] Harmony T. (2013). The functional significance of delta oscillations in cognitive processing. Front. Integr. Neurosci. 7:83. 10.3389/fnint.2013.00083 24367301 PMC3851789

[B28] Hong S. L. (2010). The entropy conservation principle: application in ergonomics and human factors. Nonlinear Dynamics Psychol. Life Sci. 14, 291–313. 20587303

[B29] Hubbard J. Gent T. C. Hoekstra M. M. B.. (2020). Rapid fast-delta decay following prolonged wakefulness marks a phase of wake-inertia in NREM sleep. Nat. Commun. 11:3130. 10.1038/s41467-020-16915-0 32561733 PMC7305232

[B30] Kaufman M. T. Churchland M. Ryu S. I. Shenoy K. V. (2015). Vacillation, indecision and hesitation in moment-by-moment decoding of monkey motor cortex. eLife 4:e04677 10.7554/eLife.04677 25942352 PMC4415122

[B31] Kennedy T. Regehr G. Baker G. R. Lingard L. A. (2005). Progressive independence in clinical training: a tradition worth defending? Acad. Med. J. Assoc. Am. Med. Coll. 80, S106–11. 10.1097/00001888-200510001-00028 16199447

[B32] Klimesch W. (2012). Alpha-band oscillations, attention and controlled access to stored information. Trends Cogn. Sci. 16, 606–617. 10.1016/j.tics.2012.10.007 23141428 PMC3507158

[B33] Klimesch W. Sauseng P. Hanslmayr S. (2006). EEG alpha oscillations: the inhibition-timing hypothesis. Brain Res. Rev. 53, 63–88. 10.1016/j.brainresrev.2006.06.003 16887192

[B34] Knill D. C. Pouget A. (2004). The bayesian brain: the role of uncertainty in neural coding and computation. Trends Neurosci. 27, 712–719. 10.1016/j.tins.2004.10.007 15541511

[B35] Kothe C. (2014). Lab Streaming Layer (LSL). Available online at: https://github.com/sccn/labstreaminglayer (accessed January 14, 2021).

[B36] Lachaut J. P. Axmacher N. Mormann F. Halgren E. Crone N. E. (2012). High-frequency neural activity and human cognition: past, present and possible future of intracranial EEG research. Prog. Neurobiol. 98, 279–301. 10.1016/j.pneurobio.2012.06.008 22750156 PMC3980670

[B37] Lachaux J. P. Jung J. Dreher J. C. Bertrand O. Minotti L Hoffman D.. (2008). Silence is golden: transient neural deactivation in the prefrontal cortex during attentive reading. Cerebral Cortex 18, 443–450. 10.1093/cercor/bhm085 17617656

[B39] Likens A. D. Amazeen P. Stevens R. Gorman J. Galloway T. (2014). Neural signatures of team coordination are revealed by multifractal analysis. Soc. Neurosci. 9, 219–234. 10.1080/17470919.2014.882861 24517441

[B38] Likens A. D. Wiltshire T. J. (2020). Windowed multi-scale synchrony: Modeling time-varying and scale-localized interpersonal coordination. Soc. Cogn. Affect. Neurosci. 30:nsaa130 10.1093/scan/nsaa130 PMC7812625 32991716

[B40] Maudrich T. Kenville R. Maudrich D. Villringer A. Ragert P. Nikulin V. V. (2020). Voluntary inhibition of physiological mirror activity: an EEG-EMG study. eNeuro 7:ENEURO.0326-20.2020. 10.1523/ENEURO.0326-20.2020 33055200 PMC7598909

[B41] McKiernan K. A. Kaufman J. N. Kucera-Thompson J. Binder J. R. (2003). A parametric manipulation of factors affecting task-induced deactivation in functional neuroimaging. J. Cogn. Neurosci. 15, 394–408. 10.1162/089892903321593117 12729491

[B42] Mullen T. (2012). NITRC: CleanLine: Tool/Resource Info. Available online at: http://www.nitrc.org/projects/cleanline (accessed January 14, 2021).

[B43] Mullen T. R. Kothe C. A. Chi Y. M. Ojeda A. Kerth T. Makeig S.. (2015). Real-time neuroimaging and cognitive monitoring using wearable dry EEG. IEEE Trans. Biomed. Eng. 62, 2553–2567. 10.1109/TBME.2015.2481482 26415149 PMC4710679

[B44] Murray J. Bernacchia A. Freedman D. Romo R. Wallis J. D. Cai X.. (2014). A hierarchy of intrinsic timescales across primate cortex. Nat. Neurosci. 17, 1661–1663. 10.1038/nn.3862 25383900 PMC4241138

[B45] Neubauer A. C. Fink A. (2009). Intelligence and neural efficiency. Neurosci. Biobehav. Rev. 33, 1004–1023. 10.1016/j.neubiorev.2009.04.001 19580915

[B46] Nikolaus K. Marieke M. Peter B. (2008). Representational similarity analysis - connecting the branches of systems neuroscience. Front. Syst. Neurosci. 2:4 10.3389/neuro.06.004.2008 19104670 PMC2605405

[B47] Oostenveld R. Fries P. Maris E. Schoffelen M. (2011). FieldTrip: open source software for advanced analysis of MEG, EEG, and invasive electrophysiological data. Comput. Intell. Neurosci. 2011:156869. 10.1155/2011/156869 21253357 PMC3021840

[B48] O'Rielly T. X. (2013). Making predictions in a changing world: inference, uncertainty and learning. Front. Neurosci. 7:105. 10.3389/fnins.2013.00105 23785310 PMC3682109

[B49] Ossandon T. Jerbi K. Vidal J. R. Bayle D. J. Henaff M. A. Jung J.. (2011). Transient suppression of broadband gamma power in the default mode network is correlated with task complexity and subject performance. J. Neurosci. 31, 14521–14530. 10.1523/JNEUROSCI.2483-11.2011 21994368 PMC6703400

[B50] Perez A. Dumas G. Karadag M. Dunabeitia J. A. (2019). Differential brain-to-brain entrainment while speaking and listening in native and foreign languages. Cortex 111, 303–315. 10.1016/j.cortex.2018.11.026 30598230

[B51] Pezzulo G. Rigoli F. Friston J. J. (2018). Hierarchical active inference: A theory of motivated control. Trends Cogn. Sci. 22:4. 10.1016/j.tics.2018.01.009 29475638 PMC5870049

[B52] Rumelhart D. E. (1980). “Schemata: the building blocks of cognition,” in Theoretical Issues in Reading Comprehension (Perspectives from Cognitive Psychology, Linguistics, Artificial Intelligence and Education), eds Spiro R. J Bruce B. C. Brewer W. F. (Mahwah, NJ: Erlbaum), 33–58. 10.4324/9781315107493-4

[B53] Sänger J. Müller V. Lindenberger U. (2012). Intra- and interbrain synchronization and network properties when playing guitar in duets. Front. Hum. Neurosci. 6:312. 10.3389/fnhum.2012.00312 23226120 PMC3509332

[B54] Schank R. C. Abelson R. P. (1977). Scripts, Plans, Goals and Understanding: An Inquiry into Human Knowledge Structures. Mahwah, NJ: Lawrence Erlbaum Associates.

[B55] Schneider D. W. Logan D. (2015). Chunking away task-switch costs: a test of the chunk-point hypothesis. Psychol. Bull. Rev. 22:884–889. 10.3758/s13423-014-0721-3 25214458 PMC4362891

[B56] Sedley W. Cunningham M. O. (2013). Do cortical gamma oscillations promote or suppress perception? An under-asked question with an over-assumed answer. Front. Hum. Neurosci. 7:595. 10.3389/fnhum.2013.00595 24065913 PMC3778316

[B57] Sengupta B. Stemmler M. B. Friston K. J. (2013). Information and efficiency in the nervous system—a synthesis. PLoS Comput. Biol. 9:e1003157. 10.1371/journal.pcbi.1003157 23935475 PMC3723496

[B58] Shannon C. (1948). The mathematical theory of communication. Bell Syst. Tech. J. 27, 379–423. 10.1002/j.1538-7305.1948.tb01338.x

[B59] Shannon C. E. (1951). Prediction and entropy of printed English. Bell Syst. Tech. J. 30, 50–64. 10.1002/j.1538-7305.1951.tb01366.x

[B60] Shipp S. (2016). Neural elements for predictive coding Front. Psychol. 7:1792. 10.3389/fpsyg.2016.01792 27917138 PMC5114244

[B61] Soltani A. Izquierdo A. (2019). Adaptive learning under expected and unexpected uncertainty. Nat. Rev. Neurosci. 20, 435–544. 10.1038/s41583-019-0180-y 31147631 PMC6752962

[B62] Stephens G. Silbert L. Hasson U. (2010). Speaker-listener neural coupling underlies successful communication. Proc. Natl. Acad. Sci. U.S.A. 107, 14425–14430. 10.1073/pnas.1008662107 20660768 PMC2922522

[B63] Stevens R. Galloway T. (2014). Toward a quantitative description of the neurodynamic organizations of teams. Soc. Neurosci. 9, 160–173. 10.1080/17470919.2014.883324 24502273

[B64] Stevens R. Galloway T. (2015). Modeling the neurodynamic organizations and interactions of teams. Soc. Neurosci. 11, 123–139. 10.1080/17470919.2015.1056883 26079050

[B65] Stevens R. Galloway T. (2016). Tracing neurodynamic information flows during teamwork. Nonlinear Dynamics Psychol. Life Sci. 20, 271–292. 27033135

[B66] Stevens R. Galloway T. (2017). Are neurodynamic organizations a fundamental property of teamwork? Front. Psychol. 8:644. 10.3389/fpsyg.2017.00644 28512438 PMC5411457

[B67] Stevens R. Galloway T. (2019). Teaching machines to recognize neurodynamic correlates of team and team member uncertainty. J. Cogn. Eng. Decis. Making 13, 310–327. 10.1177/1555343419874569

[B68] Stevens R. Galloway T. Halpin D. Willemsen-Dunlap A. (2016). Healthcare teams neurodynamically reorganize when resolving uncertainty. Entropy 18:427, 10.3390/e1812042728512438

[B69] Stevens R. Galloway T. Lamb J. Steed R. Lamb C. (2017). “Linking team neurodynamic organizations with observational ratings of team performance,” in Innovative Assessment of Collaboration, eds Von Davier A. A. Kyllonen P. C. Zhu M. (Cham: Springer International Publishing), 10.1007/978-3-319-33261-1_20

[B70] Stevens R. Galloway T. Willemsen-Dunlap. (2018). Quantitative modeling of individual, shared and team neurodynamic information. Hum. Factors 60, 1022–1034. 10.1177/0018720818781623 29906201

[B71] Stevens R. Galloway T. L. Willemsen-Dunlap A. (2019). Advancing our understandings of healthcare team dynamics from the simulation room to the operating room: a neurodynamic perspective. Front. Psychol. 10:1660. 10.3389/fpsyg.2019.01660 31456706 PMC6699601

[B72] Stevens R. Gorman J. C. Amazeen P. Likens A. Galloway T. (2013). The organizational neurodynamics of teams. Nonlinear Dynamics Psychol. Life Sci. 17, 67–86. 23244750

[B73] Stroulia E. Goel A. K. (1994). Task structures: what to learn? AAAI Tech. Rep. SS94–02, DD94–DD02.

[B74] Sun C. Yang W. Martin J. Tonegawa S. (2020). Hippocampal neurons represents events as transferable units of experience. Nat. Neuro. 23, 651–663. 10.1038/s41593-020-0614-x 32251386 PMC11210833

[B75] Tognoli E. Kelso J. A. (2015). The coordination dynamics of social neuromarkers. Front. Hum. Neurosci. 9:563. 10.3389/fnhum.2015.00563 26557067 PMC4617382

[B76] Walker E. Y. Cotton R. J. Ma J. W. Tolias A. S. (2020). A neural basis of probabilistic computation in visual cortex. Nat. Neurosci. 23, 122–129. 10.1038/s41593-019-0554-5 31873286

[B77] Welch P. (1967). The use of fast fourier transform for the estimation of power spectra: a method based on time averaging over short, modified periodograms. IEEE Trans. Audio Electroacoustics 15, 70–73. 10.1109/TAU.1967.1161901

[B78] Wianda E. Ross B. (2019). The roles of alpha oscillations in working memory retention. Brain Behav. 9:e01263. 10.1002/brb3.1263 30887701 PMC6456781

[B79] Yon D. Zainzinger V. de Lange F. P. Eimer M. Press C. (2020). Action biases perceptual decisions toward expected outcomes. J. Exp. Psychol. Gen. 10.1037/xge0000826 33289575 PMC8515773

[B80] Zénon A. Solopchuk O. Pezzulo G. (2018). An information-theoretic perspective on the costs of cognition. Neuropsychologia 123, 5–18. 10.1016/j.neuropsychologia.2018.09.013 30268880

[B81] Zhang L. Qiu F. Zhu H. Xiang M. Zhou L. (2019). Neural efficiency and acquired motor skills: an fMRI study of expert athletes. Front. Psychol. 10:2752. 10.3389/fpsyg.2019.02752 31866917 PMC6908492

